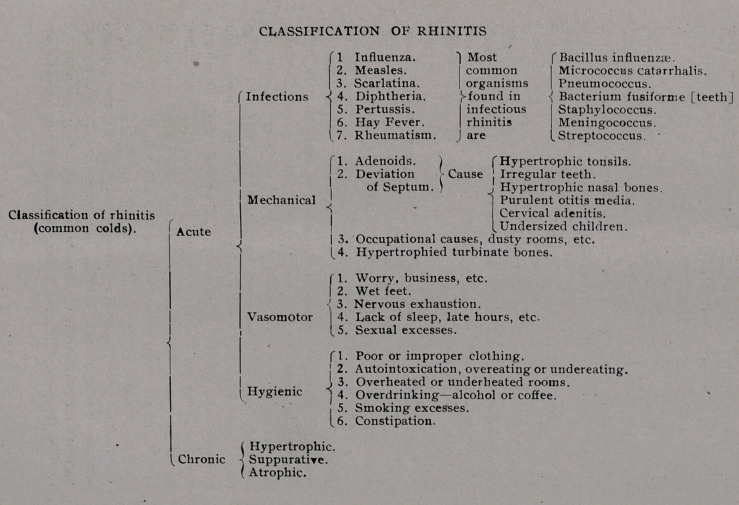# Selections and Abstracts

**Published:** 1914-04

**Authors:** 


### SELECTIONS AND ABSTRACTS

### PRELIMINARY PROGRAM AMERICAN PROCTOLOGIC SOCIETY.

Sixteenth Annual Meeting, Atlantic City, N. J.

Commencing Monday, June 22, 1914.

Executive Council meets at 11 A. M.

First Regular Session at 2 P. M.

Annual Address of the President—

Subject: The Future of Procto-Enterology. Joseph M. Mathews, Louisville, Ky.

PAPERS

1—A Review of Proctologic Literature for 1913. Samuel T. Earle, Baltimore, Md.

2—Abnormalities of the Colon, as seen with the Roentgen Ray; Lantern Slide Demonstration. Walter Irwin LeFevre, Cleveland, Ohio.

3—Coccygodynia: A New Method of Treatment by Injections of Alcohol. Frank C. Yeomans, New York City, N. Y.

4—The Technique of the Perineal Operation for Cancer of the Rectum. Jas. A. MacMillan, Detroit, Mich.

5—Myasthenia Gastro-Intestinalis. V. Lee Fitzgerald, Providence, R. I.

6—Further Observations on the Treatment of Pruritis Ani by Autogenous Vaccines. Dwight H. Murray, Syracuse, N. Y.

7—A Report of Cases of Pruritus Ani Treated with Carnotite. Saml. T. Earle, Baltimore, Md.

8—Treatment of Amoebic Dysentery with Emetin Hydrochloride. Alfred J. Zobel, San Francisco, Cal.

9—Amoebic Dysentery and its Treatment. William M. Beach, Pittsburg, Pa.

10—Some Consideration of Colonic Surgery. Louis J. Hirschman, Detroit, Mich.

11—Myxorrhea Coli, Membranacea and Colica. Saml. G. Gant, New York City, N. Y.

12—Hemorrhagic Colitis; with Report of Three Cases. Jerome M. Lynch, New York City, N. Y.

13—Retro-Rectal Gumma; Report of Two Cases. Alois B. Graham, Indianapolis, Ind.

14—Anal and Rectal Growths of Benign or Doubtful Character. T. Chittenden Hill, Boston, Mass.

15—Retro-Rectal Infections. Collier F. Martin, Philadelphia, Pa.

16—Radium, Its Use in Proctology. J. Rawson Pennington, Chicago, Ill.

17—Rectal Adenomata. Granville S. Hanes, Louisville, Ky.

18—Hyperplastic Tuberculosis of the Colon. J. M. Frankenburger, Kansas City, Mo.

19—Pseudo Intestinal Stasis and Real Intestinal Stasis Demonstrated Roentgenologically. Arthur F. Holding, New York City, N. Y.

20—Local Treatment of Anal Fissure. Jas. A. Duncan, Toledo, Ohio.

21—Reflex Symptoms Arising in the Rectum and Anus. George B. Evans, Dayton, Ohio.

22—Some Unusual Results of Sigmoidoscopy. Ralph W. Jackson, Fall River, Mass.

23—Crude and Careless Diagnostic Methods: Results of, in Reported Cases of Recto-Colonic Conditions. John L. Jelks, Memphis, Tenn.

### THE MAGGOT TRAP A POSSIBLE SOLUTION OF THE FLY PROBLEM.

### U. S. Department of Agriculture Makes Successful Experiments That Should Be of Interest to Health Officers and Sanitarians.

A trap to destroy the maggots of the typhoid or housefly before they develop into winged insects is a possible solution of the fly problem and one that should interest health officers, sanitarians and others who might make use of it on manure heaps where this common pest breeds. The Department of Agriculture's scientists in their preliminary experiments with such a trap have succeeded in destroying from 70 to 99 per cent of the maggots in a pile of manure. This method of attack differs from those which have been generally used. Fly poisons have been made to tempt the appetite of the adult fly, or fly-tight receptacles have been used to keep the adult female from laying her eggs in manure. The newer method is based on the knowledge of certain habits of the undeveloped fly maggot.

The maggots of the typhoid fly, it has been discovered, have a habit of migrating from their breeding places into drier portions of the manure heap. This seems a distinct move on their part to permit the adult fly to issue from the refuse in the easiest and quickest manner. The efficiency of the new trap is based on the regularity of this deep-seated habit.

### DESCRIPTION OF A MAGGOT TRAP.

A large galvanized iron pan, measuring 5 by 3 feet, with sides 4 inches high, was made. In this stood a container on legs 8 inches high. This container measured 4 by 2 by 2 feet. The sides and bottom were of heavy wire, ¼-inch mesh, supported by a light wooden framework. Twelve cubic feet of manure well infested with eggs and larvae were placed in this container and sprinkled with water. Water was also poured into the pan below to the depth of about 1 inch. Surrounding and covering both pan and container was a fly-tight inclosure made of a large cage, 6 by 6 by 6 feet. This prevented further infestation of the manure, and an arrangement of traps at the top of the cage made it possible to capture and keep a record of any flies that might emerge. At the time for the emergence of flies the sides of the cage were darkened with black cloth in order to drive the flies into the traps at the top. Each day the maggots were collected from the pan and counted, and each day the manure in the container was sprinkled thoroughly with water and the pan was washed out and again partly filled with water to drown the larvae which fell into it.

The experiments of the Department's entomologists showed that from 98 to 99 per cent of all the maggots in the manure pile were destroyed, if the manure was kept moist. From comparatively dry manure about 70 per cent. were destroyed.

These experiments, as yet, have been tried only on a comparatively small scale. The question immediately arises whether the trap which appears so successful on a small scale can be adapted to the handling of manure in a practical way on a large scale. Every consideration points to the probability that it can and that it will afford "an additional weapon of great value." However, the final verdict as to the value of the maggot trap must wait upon the solution of certain practical problems. To point out some of these here is to suggest lines for further investigation.

(2) In the first place, here must be determined what form, size, and construction of trap will give the best results. The answer to this will depend largely on the particular conditions obtaining at any given stable, such as the amount of manure produced daily, the arrangements for drainage, etc. It will also depend on the answer to the following problems:

(2) How deeply may manure be heaped in a trap without interfering with the migration? It will probably be found that the depth will make little difference, provided that the manure is kept moist, and provided that avenues of escape are afforded at the sides as well as at the bottom.

(3) How long must manure be kept in a maggot trap before it is entirely free from larvae? This is very important question from a practical standpoint, and one will find scant suggestion as to the answer in the literature on the life history and habits. The housefly breeds preferably in horse manure, but it has never determined just how long a given lot of manure continues to be an attractive place for egg laying, nor for how long a period fly larvae will continue to appear in it. It is obvious that the maggot trap would not be practical if the infestation of the manure were daily renewed for a long time. Under ordinary conditions the drying of the surface of a heap of manure probably limits the peroid of egg laying to the first day or two of exposure. But in a maggot trap the manure must be kept wet in order to insure the greatest amount of migration. Would not such a moist surface be daily reinfested and maggots continue to appear in the manure as long as any fermentation were in progress? As a matter of fact, the period of infestation appears to be rather short, and even under the most favorable conditions maggots will rarely be found in a given lot of manure after 10 or 12 days' exposure. In support of this claim some experimental data may be given here.

(4) The disposal of the maggots is another practical consideration. If the larvae were allowed to drop to the ground they would burrow into it to pupate there and nothing would be gained. It would be necessary to have some sort of vessel, e. g., a concrete basin, beneath the trap. This should have vertical sides and contain an inch or more of a weak disinfectant or of water covered with a film of kerosene oil. If such a basin were connected with a sewer or cesspool the maggots collecting in it could be flushed out each week without the necessity of handling them in any way and without any offensive decomposition.

That the maggot trap possesses certain advantages is obvious and ought to lead to many attempts to develop it along practical lines. Cheapness would be one of its strong points. Practically the only cost would be the initial one for the construction of the trap and of a basin or receptacle for catching and disposing of the maggots. Very little additional time or labor would be required in operating it. The sprinkling of the manure would be a very small part of the daily routine of removing the manure from the stables. Proper arrangements for the disposal of the maggots would require only a few minutes' attention at long intervals.

**Greater details** of the experiments with the maggot trap are given in the U. S. Department of Agriculture's new bulletin entitled: "The Migratory Habit of Housefly Larvae as Indicating a Favorable Remeddial Measure. An Account of Progress." Many scientists now prefer to call the housefly the "typhoid fly" or the "manure fly" because of the real danger that lurks in this widely distributed pest. This fly is one of the most active agents in spreading typhoid fever. It spreads Asiatic cholera and other diseases of the intestines. It has even been known to be a minor factor in spreading tuberculosis. Its chief breeding place is the manure heap from which it may fly directly into the house.

---

### THE SIMULATION BY MALARIA OF ACUTE SURGICAL ABDOMINAL DISEASE, ESPECIALLY APPENDICITIS.

By Walter M. Brickner, M. D.

Adjunct Surgeon, Mount Sinai Hospital; Surgeon, Philanthropin Hospital, New York.

The mistake is often made of jumping to the diagnosis of malaria in cases of surgical infection marked by chill and sudden pyrexia. It is no part of my intention to encourage or excuse this error, which must find its only support in the well-established observation that surgical operations and the puerperal state do, indeed, occasionally light into activity a latent paludism.

It is my purpose to call attention to the opposite mistake, viz, the treatment as an acute surgical lesion, particularly appendicitis, of a malarial seizure marked by localized abdominal symptoms.

The occasional occurrence of these abdominal manifestations in malaria is but little recognized in text-books and monographs dealing with the disease. Thus, abdominal pain as a symptom is not mentioned in "Traite du Paludisme" by Laveran himself or in the "Practice of Medicine" by Osler, in whose clinic also extensive original studies of malaria had been made. In Thayer's "Lectures on the Malarial Fevers" considerable space is devoted to differential diagnosis, but no abdominal affection is there included—except typhoid fever. In the standard works on appendicitis by Deaver and by Kelly, I have looked in vain through the long sections on differential diagnosis for any reference to malaria.

However, the simulation of appendicitis by malaria is not unknown to our literature. In Craig's excellent work on "The Malarial Fevers" (1909) occurs the following definite statement (page 189): "Pain over the stomach and abdomen is not infrequently met with and has been the cause of many mistaken diagnoses. I have repeatedly seen cases of malaria with severe pain in the region of the appendix diagnosed as appendicitis, and in more than one instance the microscope saved the patient an operation. In some cases there is general pain over the abdomen simulating very closely that of general peritonitis. I have observed instances in which the abdominal pain was agonizing in character, and controlled only by large doses of morphine."

Here and there one encounters reports of specific instances. Thus, in 1902 Henry Wolf, of New York, to whom especial credit belongs for calling attention to the possible confusion of these two affections, recorded a case of malaria simulating appendicitis, and a case of malaria coexisting with appendicitis, as proved operatively and hematologically. As a more recent instance, Graham Henson, of Florida, briefly described a case of malaria in which the seizures were marked by all the symptoms and signs of appendicitis, including muscle rigidity. And, too, one occasionally hears of an unrecorded case in which an innocent appendix has been hastily removed and the surgeon has been embarrassed a day or two later by the occurrence of a chill and the finding of plasmodia.

The following cases from my own experience will amply illustrate the condition under discussion:

Case I.—On the evening of October 12, 1910, a colleague telephoned to me that he had sent into the private ward at Mt. Sinai Hospital a patient with either acute appendicitis or acute cholecystitis, of two days' standing, and he desired me to operate upon her at once. I found a rather stout woman of 50, with a temperature of 103 deg. F., pulse 100, who had during the day increasingly severe pain in the epigastrium, hypogastrium, and back, and at 4 P. M. a slight chill, rise of temperature to 103.8 deg. F., and vomiting. At 8 P. M., when I saw her, she was still nauseated and she was suffering considerable abdominal pain, especially on the right side. The abdomen was not rigid; but there was tenderness in the right hypochondrium, where the edge of the liver was palpable, marked tenderness in the right iliac region, where the spontaneous pain was greatest, and slight tenderness in the left iliac region. The tip of the spleen was palpable. Vaginal and thoracic examinations were negative. The case made upon me the general impression that it was not one of cholecystitis or appendicitis; and the suggestion afforded by the enlarged spleen made me hazard the diagnosis of malaria with abdominal symptoms. Both the family physician and the house surgeon—astute and capable men—were horrified at my objection to an immediate operation, especially when the blood examination, then made, showed a leukocytosis of 20,000, 86 per cent. polynuclears and no plasmodia. The family physician urged as an additional argument in favor of appendicitis or gall-stone disease the fact that the patient had had a similar attack of pain and vomiting one month before. But the history of that attack and the other anamnestic data, which I pieced together that night and the following day, only served to confirm the opinion afforded by my physical examination. These data were as follows:

The patient had had one "attack of appendicitis with peritonitis" (?) and one "attack of gall-stones" (?), each several years before, neither submitted to operation. She never had typhoid. In August she had been in Port Washington, L. I., and in New Haven. In September she had the attack of abdominal pain referred to by her physician, accompanied by severe vomiting of sudden onset at 4 P. M. But the temperature had risen to 105 deg. F., and the attack lasted but one day. On October 10th, at 4 P. M., she had a chill, followed by fever and sweating, abdominal pain and backache. On October 11th the temperature was 99 deg. F. in the morning, but at 6 P. M. there were again chill, fever, pain and vomiting. On October 12th, the day of admission, the chill was again at 4 P. M. This alternation indicated, if my diagnosis were correct, a double tertian (quotidian) infection—and so it proved to be.

During the night the abdominal symptoms subsided somewhat, and on October 13th the temperature was 99 deg. F all day, there was no pain and the tenderness was less. At 8 P. M. the temperature rose to 106 deg. F., there was a sharp chill, and numerous plasmodia were found. On October 14th the seizure again came at 4 o'clock. After that date, under quinine administration, there were no attacks, and throughout the period that the patient remained in the hospital there was no return of the abdominal symptoms.

Case II.—On April 18, 1913, another very capable and careful practitioner referred to me for operation a youth of 16 as a case of acute appendicitis. Four months before he had had an acute attack diagnosed appendicitis—abdominal pain, right iliac tenderness, vomiting, fever and short chill. He was well after that until the day before I saw him (April 17th), when he was again seized with severe abdominal pain localizing in the appendix region, vomiting, chilliness, fever (101 deg. F.) and marked local tenderness, without rigidity. On the morning of April 18, when seen by his physician, his temperature was 100 deg. F., pulse 100, he had right iliac pain and very marked local tenderness. When I saw him at 11:30 A. M. his face was very pale, of a yellow-gray color, he was distinctly chilly and had been so, he said, for twenty minutes, his temperature was 104.2 deg. F. and his pulse 130. His abdomen was soft, but the right iliac region, where he was having severe pain, was very tender. There was no area of hyperesthesia; the rectal examination was negative; Meltzer sign negative.

The very high temperature, slight chill and very rapid pulse indicated either that the condition was not appendicitis or that, if appendicitis, it was associated with mesenteric or portal vein thrombosis, which latter neither the facial appearance nor the soft abdomen supported. In spite of the fact that the spleen was not enlarged to percussion or palpation, this case also impressed me as a probable malaria; and after a prolonged study of the fresh blood I found a very few plasmodia. In the hour or so that the patient and his blood were being examined, his chill ceased, his color greatly improved and his temperature fell to 103 deg. F.

At his physician's request the boy was admitted to the Philanthropin Hospital for futher observation. That afternoon his temperature fell to 101 deg. F., his color returned, the abdominal signs and symptoms disappeared completely, and he became quite comfortable. His blood then showed 18,000 leucocytes per cub. mm. and 76 per cent. polynuclears, and a stained smear made in the morning also showed a few malarial parasites.

Throughout the next day (April 19th) the temperature was 101 deg. F., pulse 100. No symptoms. April 20th, temperature normal, feels perfectly well. April 21st, temperature normal, spleen still not palpable. Quinine begun.

Case III.—In the summer of 1911 I was called to Rockaway Beach to operate upon a young woman who had been seized with right iliac pain and tenderness, vomiting and fever. She had been operated upon for appendicitis some time before and it was presumed that part of the organ had not been removed. When I reached her, a couple of hours later, she had, indeed, considerable tenderness at the site of the scar, but her temperature had risen to 106 deg. F., and her spleen was easily palable. Although she had no distinct chill the diagnosis of malaria (subsequently corroborated by blood examination) was obvious, and I refused to operate. Her febrile symptoms disappeared under quinine, but the abdominal symptoms continued, and she was operated upon a few weeks later for perityphlic adhesions.

Here is a case of quotidian fever simulating appendiceal or gall-bladder disease, a case of irregular malaria simulating acute appendicitis, and a rather straightforward malarial attack associated with intestinal adhesions—in all three of which cases competent practitioners had urged immediate operation. What, then, are the differentiating features by which we may recognize the occasional instances of malaria simulating acute surgical abdominal lesions?

Abdominal pain, as we have seen, may occur in malaria; it may be localized, especially in the right iliac region; there may be distinctly localized and marked tenderness; and Henson's case indicates that there may even be rigidity.

I do not know whether localized hyperesthesia may be present in the malaria attack; I do know that it may be absent in appendicitis and gall-bladder disease.

Meltzer's sign, when present, is helpful in diagnosing appendicitis; but, of course, one must determine that it is not also on the left side. Perhaps, too, it may be found in some of these malarial cases with right iliac signs.

Pain in the back is very common in paludal seizures; it is also common in gall-stone attacks; it is not common in appendicitis, but pain in the right lumbar region often accompanies appendicitis, especially the retrocecal variety.

Vomiting is often a pronounced feature of the malarial attack, as it is in appendicitis and other abdominal diseases.

The fever may, indeed, direct our attention towards the possibility of malaria. But in the latter the temperature rise may be moderate throughout, or at the time of observation. On the other hand, a temperature of 104 or even 105 deg. F. may be seen in empyema or gangrene of the gall-bladder—which, however, usually give fairly definite signs—or in cholangitis, with which there is more or less jaundice (which latter may also occur in remittent fever.) Very high temperature is uncommon in appendicitis, but a temperature of 103 deg. F. may mark an empyema of the appendix; while a temperature of 104 deg. F.

may be found with appendiceal abcess or purulent peritonitis, the physical signs of which (mass, abdominal contour, or peculiar character of the rigidity) are usually fairly distinctive. A still higher temperature may exist in appendicitis when there is mesenteric or portal vein thrombosis.

A chill, too, is suggestive of a possible malaria. But in that disease the chill may be absent or slight, throughout or at the time of examination. A pronounced chill is not at all uncommon, on the other hand, in gall-bladder or gall-duct suppuration; a moderate chill is not rare in appendicitis, and there may be a distinct chill in beginning peritonitis, in appendiceal empyema or, especially, in invasion of the mesenteric veins.

An enlargement of the spleen is suggestive of malaria; but it may be absent, as in case II, or due to an old malaria, a recent typhoid or other cause.

If the generally accepted teaching were entirely correct, the blood count could be depended upon to establish at once a differential diagnosis, since a leukocytosis is usually supposed to exclude malaria. Thus Thayer says "The presence of an appreciable leukocytosis is strong evidence against the existence of an uncomplicated malarial fever." That this cannot be relied upon is demonstrated in two of my cases, one of which showed 20,000 and the other 18,000 leukocytes, per cub. mm. Here, again, we find illuminating information in Craig's work (pages 131-2): "In acute malarial infection the leukocytes are reduced in number, both absolutely and relatively to the red blood cells. Thus the malarial fevers . . . are characterized, as a rule, by a marked leukopenia, only the pernicious forms showing a leukocytosis. During the first hours of an acute attack of any of the forms of malaria there may be a more or less marked leukocytosis, sometimes only visible during the first fifteen or twenty minutes, at others still demonstrable until the decline of the fever. Generally the leukocytosis is only observable for a short time at the onset of the paroxysm, soon giving place to the characteristic leukopenia." The blood count, then, is helpful but not diagnostic, since a leukocytosis may be absent in appendicitis and present in malaria.

The finding of plasmodia is, of course, diagnostic of malaria; but they may be apparently absent at the time of observation. And, too, as in Wolf's case, malaria and appendicitis may rarely coexist—in which case, no doubt, one would be justified in withholding operation for a few hours or more of observation to determine the relative seriousness of the two diseases.

If, then, we exclude the finding of plasmodia—which, indeed, one is not apt to discover unless other signs or symptoms suggest a patient search for them—there is not a single differentiating feature that can here replace the clinical sense, the "sizing-up" of a case in the light of experience; or, to be somewhat more specific, the study of the facial expression and the "abdominal expression," and the balancing of physical signs with subjective symptoms and the anamnestic data.

In one of Wolf's cases and in one of mine there was a coexisting lesion in the right iliac fossa. We have no reason to believe, however, that this is often the case; and I am quite unable to suggest an explanation for the occasional association with malarial attacks of localized pain and tenderness at the appendix site.

I must content myself, therefore, in this clinical discourse, with the observation that malaria is not rare in New York City, especially in the Long Island and Staten Island sections and in that portion of the Bronx bordering on the Sound; and with emphasizing that malaria, like pneumonia and pleurisy, is one of the extra-abdominal affections that should be excluded before operating in a case of supposed surgical abdominal disease.

---

### THE PERNICIOUS NASAL PACKING

Custom dies hard, whether it be in the realm of manners, religion or surgery. One of the most unaccountable survivals of a vanishing past is the custom of packing a nasal cavity after a turbinectomy. This absolutely gratuitous insult to inoffensive and unoffending tissues can be indicted upon four counts, viz., unnecessary precautions against hemorrhage, unnecessary infliction of pain upon a patient in an operation that ought to be painless, danger of sepsis in a region communicating with the meninges, and the danger of secondary hemorrhage.

That packing is an unnecessary precaution against hemorrhage is readily seen from a consideration of the field operated upon and from the results of practice. The vessels cut across are all small vessels, and, at the time the section is finished, are under the control of the cocaine and synthetic suprarenin that have been used for anesthesia and hemostasis. The sitting posture favors the return venous flow to the heart, and reduces the intravascular pressure to a minimum. Inspiration through the nose and expiration through the mouth favors the formation of clots in the mouths of the divided vessels—and air is one of our most efficient hemostats. When the operation has been a clean one, when no shreds and tags with only partly divided vessels have been left behind and the measures indicated above have been carried out, there is no danger of hemorrhage, and the customary packing of the nasal chamber with gauze is unnecessary and cruel.

Cruel it is, because if it is done at all it should be done tightly enough to directly control any theoretical flow of blood from the divided vessels, and this tight packing engenders severe pain, which disturbs sleep and renders the patient miserable until the packing is taken out, one or two days later.

This tightness of the packing brings us to the third reason why packing after turbinectomy should never be done. In the first place, we are never able to attain perfect asepsis in the nose. Although we may douche the lower meati, bacteria-laden secretions from one or more of the accessory sinuses are certain to enter the nasal cavity. The firmly packed gauze not only irritates the delicate mucous membrane of the naris, but actually erodes it, and when the packing is removed innumerable denuded areas may be seen, each one the site of a slough or a favorable portal of infection from the blood and mucus-stained gauze.

Clinically, the swollen face of the patient bears evidence to the obstructed circulation, hemic and lymph, and to the irritation of the nasal peripheral nerves. The damage done to the tissues by the gauze has been far greater than that inflicted by the cutting instruments at the time of operation. Signs such as follow the packing of the nasal cavities—cavities with an intimate vascular connection with the orbit and the interior of the cranium—ought to arrest the attention and the caution of the rhinologist and lead to the abandonment of this pernicious practice.

Finally, the unnecessary gauze packing does not attain the object for which it was designed. There would be nc hemorrhage from the cut surface if the packing were omitted, only, at the most, a slight oozing and lasting but a few hours. But when a packing is used on account of the impossibility of using cocaine or suprarenin, the pain is severe when its removal is attempted and the removal is followed by a secondary hemorrhage that is much more profuse than the primary hemorrhage at the time of operation. And not only this, but this secondary hemorrhage is likely to recur at intervals from the packing-damaged areas, sometimes over a period of several weeks. All of these discomforts and dangers discredit the operation of turbinectomy, which, robbed of its unnecessary terrors, is a most beneficent one.

It certainly is to be hoped that the old custom, for it is nothing more than a custom dating back to the days when rhinology was young, of packing bony cavities lined with mucous membrane with gauze after an operation in anticipation of a mythical hemorrhage which never materializes, will cease to the greater comfort and safety of the patient, and the greater credit of the surgeon.—The Lancet Clinic.

### COMMON COLDS

### Their Relation to Sinus Diseases; from the Rhinologist's and the Otologist's Standpoint.

By Nathan P. Stauffer, M. D., Philadelphia

Common cold or rhinitis is an acute or chronic inflammation of the nasal or nasopharyngeal mucosa accompanied by an outpouring of serum and lymphocytes into the adjacent tissues and blood vessels. We are told that common colds were believed by Galen to be the result of a brain secretion running through the ethmoid orifices into the nares, thereby relieving the brain of the engorgement due to superfluous secretions. (Phillips.)

In the hazy past I considered all colds as just common ordinary colds, caused by wet feet and exposure; this classification I acquired through my childhood escapades and never changed, until experience in the dispensaries and private practice brought a new viewpoint. I have found every man is a law unto himself in colds. Whereas one can sit in draughts with impunity, to others of a rheumatic tendency such exposure means rhinitis, laryngitis, and bronchitis.

Nowadays I search each patient's history for the cause of his cold and base the treatment on the findings. Any reputable conscientious doctor early in his practice realizes that only douching the nose and throat is not permanently going to help his patient or his own prestige. If he desires to keep his patient's confidence, he must find the cause of the recurring rhinitis and cure it, or send the patient to some one who can. I believe the majority of colds in children start from exposure, and once the nares become swollen, the child, unable or ignorant of the way to blow his nose, soon becomes a prey to rhinitis from retained secretions, and then in turn tonsils and glands become enlarged.

I shall not endeavor to discuss exhaustively the causes, varieties of and cures of rhinitis, but briefly to point out to my readers some of the ways I classify, prevent, or cure rhinitis.

The common cold causes many diseases; it also precedes or accompanies other infections. The chart to which I ask your attention has at each heading,

I have made the following classifications of colds and their causes.

1. The chief cause, I believe, is improper clothing. You all realize how dull practice is after summer vacation, until the first cold snap catches unawares the people with light clothing, or a trip down-town without an overcoat which is sadly needed before the cool evening breeze chills one to the bone, or the April shower finds us without an umbrella or a pair of rubbers. The next day there is a stuffy feeling in the nose, or a swollen tonsil followed by the usual earache or hoarseness.

2. Repeated colds in the child, ignorant of the function of blowing his nose, are the cause of adenoids. Once adenoid tissue starts to grow, ,the posterior nares are occluded, more irritation is produced, and we have constant colds; as a mother said to me, "my baby has a fresh cold every day." This child had double otitis media, adenoids, hypertrophied tonsils, and enlarged cervical glands. Surely it had no legitimate right to hope for a cure unless the cause (adenoids) was removed. (See Fig. 1.)

3. Of all the diseases which create havoc in the nose and ear and their sinuses, I believe influenza is the worst. Next in order I should class scarlatina, measles, and hay fever; and while diphtheria, erysipelas, rheumatism, pertussis, and variola cause many grave attacks, we see these less frequently. Influenza attacks the Eustachian tube and middle ear so frequently that every winter we have epidemics of otitis media with involvement of the sinuses. Last year I had many families with many children in whom the epidemic of influenza attacked the nasal chambers and quickly traveled via the Eustachian tube to the middle ear cavity. Other organisms as the tubercle bacillus, spirochæta pallida, Leprae bacillus of Frisch in rhinoscleroma, bacillus of glanders, are sometimes found in rhinitis.

4. Worry causes loss of sleep and indigestion, then follow diminution of nerve power and a relaxation of the vasomotor system (the governing centre of the muscular coat of the arteries), then a consequent loss of resistance to infection. The following days, the mucosa weeps, a common cold is started, and medicine is of no avail if your patient cannot rid himself of his worries and get relaxation and sleep.

5. Overeating and overdrinking are usually coupled with late hours; we have in sequence a congestion, followed by auto-intoxication toxemia, with a loss of nerve force, and the mucosa again relaxes and weeps. Of what value would medicine be, if given to a man or woman who indulges in excessive whiskey or coffee drinking? They need instruction in the proper mode of living. Cure them of their gouty habits by teaching them moderation, give them exercise, and purge them; if aggravated by malformations—operate.

These are the five most common causes of rhinitis. I shall ask your attention now to the stages and discuss the treatment. Rhinitis I divide into three stages:

1. Dryness or prickling of nose and throat accompanied by chilliness. The mucosæ are quite hyperemic, dry, and free from secretions. Headache with fullness between the eyes. The temperature ranges from 99° to 104° F. Treatment comprises hot baths, purges (calomel and salts; salts especially to deplete the system of water); rest in bed; icebag to forehead; hot water bag to feet; internally ten grain Dover's powder and hot lemonade to promote sweating; in the nose, locally, epinephrine one to 5,000.

2. Profuse watery discharge, throat sore, headache less. If nose is wide, fullness between eyes diminishes, or if narrow, fullness increases. Treatment: Atropine internally until dryness of nose and throat develops; hot alkaline nasal douches; hydrochloride of cocaine daily, one per cent. solution applied. (Caution—Never prescribe cocaine for a patient to use at home). When using cocaine always have aromatic spirits of ammonia handy.

3. Mucopurulent discharge, a lowered temperature, pulse less bounding, headache diminishing unless sinuses are involved, whereupon headache and pressure symptoms increase, accompanied by dizziness. Treatment: Hot alkaline nasal douches, hydrochloride of cocaine to nares, oil sprays, strychnine sulphate, grain 1/100 three times a day, egg and milk, increased exercise, alcohol rubs twice daily; hexamethylenamine grains five, internally, three times a day.

This deals with the common cold up to the involvement of the sinuses, whether of the nose or ear, or mastoid. In considering the sinuses I ask your attention to the early symptoms, the diagnostic aids, and the surgical treatment. My experience with colds and sinus conditions has led me to conclude that the most common cause of sinus trouble is, not an acute, but a chronic mechanical condition.

Adenoids are followed by hypertrophied turbinate bones, or a diviated septum, which may be the predisposing causes; the exciting cause may be influenza, or any one of the other conditions enumerated on the chart.

A blockade of the natural drainage system of the sinuses (see figs. 2 and 4) by a bent septum and forcing the middle turbinate over, is followed by certain symptoms, which are not always as clear as those enumerated in the textbooks. The regular symptoms of sinusitis are, indefinite or localized headache; unilateral nasal discharge, with or without a slight increase of temperature; foul breath; pain, usually worse in the morning, relieved by change of position, permitting flow of pus; bone tenderness; dizziness aggravated by stooping; recurrent attacks of coryza; continual dropping of pus from posterior nares.

Eye symptoms are edema of lids, supraorbital pain, worse on reading, especially eye on side affected; deep boring pain in back of eyeballs; dread of light; distress on rotating eyeballs. To show that all sinus cases do not follow the usual course and are not so easy to diagnose, I present two cases with symptoms widely differing:

Case I. Miss T. O., aged twenty-one years, brought to the Pennsylvania Hospital with purulent otitis media and a diagnosis by family physician of acute mastoiditis. Diagnosis was confirmed and a simple mastoid operation was performed the next morning. The mastoid antrum and attic had granulations; the necrosis extended to the lateral sinus and dura but no granulations were found on either sinus or dura. Three days later, the resident told me she had chills and fever and sweats, and wanted me to open the lateral sinus; but I refused, for I remembered that it had shown no involvement at the time of the operation, also pressure now on the jugular veins failed to show the customary engorgement of the forehead veins as we should have in thrombosis of lateral sinus. I insisted on waiting a few days, and finally I was rewarded with a swollen jaw and pus in her right naris from her maxillary sinus. Here was a patient who never mentioned her jaw or nose but complained only of the inability to move her head and severe neuralgia of the ear, with headaches. Had I yielded to entreaties, I should have opened her lateral sinus on account of the typical lateral sinus symptom complex, which proved to be maxillary sinusitis complicating a mastoiditis.

CASE II. Another case will demonstrate how obscure the symptoms are to one and how plain to another. Mr. R. S., aged twenty-five years, pulse 76, temperature 99.5° F., had been treated for unilateral catarrh for a year, and suddenly manifested a small swelling on the lingual surface of the superior maxilla and was sent to his dentist, Doctor Gore, for pyorrhœa alveolaris. This dentist was an observing young man and asked if he had any unilateral nasal discharge, and upon finding a history of a right naris discharge, advised consultation so he was sent to me for examination. A deviated septum was found and pus present over anterior end of middle and inferior turbinates. Transillumination showed absence of light reflex in the right eye and darkened area over sinus. X-ray by Doctor Newcomet displayed an abscessed root, leading into the maxillary antrum, but no darkness. A Myles trocar under inferior turbinate located a large quantity of pus with a foul sickening odor. Doctor Borden extracted the second molar and I introduced a trocar into the maxillary sinus via the lingual root of the extracted molar and have syringed it out daily since; his rhinitis has ceased.

The points in this case of chronic rhinitis were the constant use of handkerchiefs, the absence of pain, of odor (noticed only by forcing air into the cavity), of tooth tenderness, or any marked symptoms pointing to his right maxillary sinus, except a slight swelling in the lingual surface of the superior maxilla, and his unilateral discharge.

The points the general practitioner wants to know are how to recognize and how to prevent sinus involvement. I should like to emphasize as suspicous: 1. Any coryza which does not tend to early recovery. 2. Any constantly recurring coryza with obstruction in nares. 3. Any unilateral discharge. 4. Headaches which are not relieved by glasses or purges.

The examination by transillumination of sinusitis with polypi and pus formation shows: 1. Absence of red pupillary reflex. 2. Absence of light over cheek and dark area under eyes. 3. No sense of light in eye when closed.

After dilatation of the nares, you can sometimes turn the head to the opposite shoulder, and pus runs out via the ostiums into nares. The x-rays are good for showing a sinus present and its outlines; but I have not found it always reliable. In two cases lately with pus in the sinuses, the x-ray did not show it to my eye.

Treatment: Of remedies cocaine one to two per cent. is the most efficient; epinephrine comes next, one to 5,000; and forty per cent. ichthyol in wool fat locally to deplete swollen mucosa, internally five grain Dover powders every three hours, salts as cathartics; ice bag to sinus; hot baths and hot nasal irrigations. Epinephrine gives more afterritation, causing more sneezing and swelling of the mucosa than cocaine. Many disagreeable symptoms are caused by cocaine, and great care should be exercised in applying it, but if you should happen to use too great strength of cocaine, give patient aromatic spirits of ammonia. People will faint from a one per cent. solution as easily as from a twenty per cent. in my experience. It seems to affect them in two ways. In the nares the irritation of rubbing it on the turbinates has many times caused patients to fall over. An excess runs down the throat and causes a choking sensation, which alarms the patient. I used to be afraid if I saw it run into the trachea, but after I saw twenty per cent. solution applied by the one half dram to the larynx for passing the bronchosope, in European and American hospitals, I ceased worrying, as I knew a little excess from a two per cent. solution would do no harm.

After thorough cocainization, I believe in douching the nose with warm saline solutions. Some object to this because of the danger of infecting the ears. I have used this for years in the Presbyterian Dispensary and also in my private practice, and I believe the good results so far outweigh the bad that I feel that it is of the greatest benefit, and I thoroughly indorse cleansing a dirty nose the same as I would any cavity containing pus. I have aborted many attacks of acute otitis media with warm nasal irrigations and an ice bag to the ear.

You have all been taught there are three turbinate bones, but, as a matter of fact, you rarely see but two and many times only the inferior. The middle and important one you can see only after cocainzing the inferior and then looking high up and to the side. To cocainize the orifices correctly, you must now slip your cocaine under the anterior end of the middle turbinate and keep gradually and gently pushing it back, allowing the cotton to remain a few minutes to give you a better view. If not, you must take a medium sized Killian speculum as Skillern advises, and inserting it under the middle turbinate, pry it outward. This may fracture the turbinae but you need not worry as this only gives better drainage. In addition to the cocainization of the natural orifices, I advise rest in bed with ice bags constantly applied to the face. It is surprising what excellent results we get with the constant ice bags and warm nasal irrigations. Calomel and saline purges help materially. If not markedly improved in ten days to two weeks, you may be sure you have a more serious condition to deal with and that brings us to the operation stage.

From the rhinologist's and otologist's standpoint I want to emphasize, in closing, that rhinitis or common cold is many times just a current attack due to:

1. Adenoids.
2. Deviations of the septum or malformations of the nose.
3. Polypi.
4. Sinus disease.

To prevent or cure rhinitis:

1. Remove the adenoids.

2. Do a submucous resection or removal of portions of the offending turbinates.

3. Snare polyps.

4. Do a Cooper, Kuster, Caldwell, Luc and Denker operation for drainage of the maxillary sinuses, or the Killian, Jansen, etc., for frontal, or the Skillern, Ballinger, Freer, or some other good surgeon's operation for ethmoid or sphenoidal sinus disease.—New York Medical Journal.

---

### ETHER OIL ANESTHESIA.

Most medical men know that at various times in the past the suggestion has been made that surgical anesthesia be produced by the introduction into the rectum of warm ether vapor. Each time the proposition has been made it has been condemned both theoretically and practically, since it has been shown that it possesses no advantages over the older method of inhalation and often causes great irritation of the bowel. Recently Gwathmey, of New York, has brought forward a newer method of inducing surgical anesthesia by the rectal injection of ether and has published his results in the New York Medical Journal of December 6, 1913. His method is a very material modification of the one we have already referred to, and consists in mixing ether with olive oil in proportions varying from equal parts to 75 per cent. to ether and 25 of oil. The oil protects the bowel or mucous membrane from the irritant effects of the ether, and he has found that olive oil is far better than any other oil for this purpose.

The patient who is to be anesthetized in this manner is prepared according to the usual method, care being taken, however, that great irritability of the bowel is not induced by the administration of a too active purge. The colon also should be thoroughly washed out until the return water is clear, and the patient should rest in bed for two hours or more before this preliminary treatment by irrigation is resorted to.

The only apparatus he employs consists of a small catheter with a funnel attached to it; two small rectal catheters inserted side by side to withdraw the fluid and irrigate the colon, and a towel which is placed over the face of the patient from time to time to prevent the dilution of the anesthetic in the air-passages as the drug is eliminated by the lungs. When the patients is satisfactorily anesthetized the towel is withdrawn. In adults he commonly employs two ounces of olive oil and six ounces of ether, the patient lying on the left side in the Sims position; a small catheter, well lubricated, is inserted three or four inches within the rectum, the funnel is attached, and the mixture poured in very slowly so that fully five minutes is expended before the injection is competed. Usually anesthesia develops in from five to twenty minutes, according to the susceptibility of the patient and the rapidity with which the injection has been given. Care must be taken that the respiratory passages of the patient are free and that breathing is properly performed with the head placed in the most advantageous position. Should any evidences of overetherization develop, as shown by signs of approaching cyanosis or embarrassed respiration, two to three ounces of the oil and ether mixture should be withdrawn by the small rectal tube, which has remained in the bowel or which can be again slipped into it. When the operator is through with his task the two small rectal tubes already mentioned are inserted as high up in the colon as possible and cold soap-suds water injected in one tube and drawn off from the other. After all the oil and ether has been abstracted in this manner 2 to 4 ounces of olive oil without ether should be introduced and the tubes withdrawn.

The advantages which Gwathmey claims for this method are that the patient has none of the disagreeable symptoms associated with inhaling this drug, that it is particularly useful in that it also is advantageous in those persons who have pulmonary diseases, as in consumptives or in those who have bronchitis and to whom an anesthetic must be given. Its contraindications are of course irritation and inflammation of the lower bowel, such as colitis, hemorrhoids, fistulæ, or malignant growth.

This method possesses certain advantages for a practitioner who has no assistant, but Gwathmey admits that in these cases the full dose had better not be given by the rectum, but the physician should give a few inhalations of ether by the lungs as well in order to carry the patient completely into unconsciousness.

Gwathmey claims that by the use of olive oil none of the objections to this method of etherization which have hitherto held exist.

Doubtless ether and oil rectal anesthesia will take its place as one of the means by which the patient can be anesthetized under extraordinary conditions. That it will supplant the inhalation method which has been so long resorted to we believe to be exceedingly unlikely.—The Therapeutic Gazette.

## Figures and Tables

**Figure f1:**